# Changes in the rate of preterm infants during the COVID-19 pandemic Lockdown Period—data from a large tertiary German University Center

**DOI:** 10.1007/s00404-023-07048-y

**Published:** 2023-05-25

**Authors:** Maria Delius, Thomas Kolben, Claudia Nußbaum, Viktoria Bogner-Flatz, Antonia Delius, Laura Hahn, Johanna Buechel, Uwe Hasbargen, Andreas W. Flemmer, Sven Mahner, Linda Hertlein

**Affiliations:** 1 Department of Obstetrics and Gynecology, LMU University Hospital, LMU Munich, Ziemssenstraße 5, 80337 Munich, Germany; 2 Division of Neonatology, Department of Pediatrics, Dr. Von Hauner Children’s Hospital, LMU University Hospital, LMU Munich, Munich, Germany; 3 Emergency Medical Services Authority, Munich, Germany; 4 World Bank, Washington, DC USA

**Keywords:** COVID-19, Lockdown, Preterm birth, Stillbirth

## Abstract

**Purpose:**

After living with the COVID-19 pandemic for more than 2 years, the impact of lockdown measures on preterm birth rates is inconsistent according to data from different countries. In this study, rates of preterm-born infants during the time of COVID-19-related lockdowns were analyzed in a tertiary perinatal center at Munich University, Germany.

**Methods:**

We analyzed the number of preterm births, infants, and stillbirths before 37 weeks of gestation during the German COVID-19 lockdown period compared to the same time periods in the years 2018 and 2019 combined. Additionally, we expanded the analysis to Pre- and Post-Lockdown Periods in 2020 compared to the respective control periods in the years 2018 and 2019.

**Results:**

Our database shows a reduction in the rate of preterm infants during the COVID-19 lockdown period (18.6%) compared to the combined control periods in 2018 and 2019 (23.2%, *p* = 0.027). This was mainly based on a reduced rate of preterm multiples during the lockdown period (12.8% vs. 28.9%, *p* = 0.003) followed by a reversed effect showing a threefold rise in multiple births after the lockdown. In singletons, the rate of preterm births was not reduced during the lockdown. The rate of stillbirths was not affected by the lockdown measures as compared to the control period (0.9% vs. 0.7%, *p* = 0.750).

**Conclusion:**

During the COVID-19 pandemic lockdown period, we found a reduced rate of preterm-born infants compared to a combined control period in the years 2018 and 2019 in our large tertiary University Center in Germany. Due to the predominant reduction in preterm multiples, we postulate that less physical activity might have led to the protective effect by lockdown measures.

## Introduction

In spring 2020, the world experienced the pandemic spread of a novel virus, known as severe acute respiratory syndrome coronavirus 2 (SARS-CoV-2) [1]. This COVID-19 pandemic has had profound effects on health-care systems, societal structures, and the world economy. The adverse effects of the COVID-19 pandemic on maternal and perinatal health are not limited to the morbidity and mortality caused directly by the disease, like a slightly higher rate of preterm births in Germany in 2020 in SARS-CoV-2-infected pregnant women according to the CRONOS register study [2]. Nationwide lockdowns, disruption of health-care services, and fear of attending health-care facilities might have also affected the well-being of pregnant women and their infants [3]. Recent evidence suggests that rates of stillbirths and preterm births might have changed substantially during the pandemic. Behavioral changes of pregnant women, as well as reduced provision of maternity services, have been discussed as possible underlying causes [4–6]. Results regarding the effects of the COVID-19 pandemic on preterm births are divergent. Studies showed an increase [7] or a decrease in the preterm birth rate [4–6, 8–11], others found no differences [12–15]. In a meta-analysis from the Lancet Global Health in 2021 discussing the effects of lockdown measures on birth outcomes, studies from high-income countries showed a decrease in numbers of preterm births, in contrast to data from low-income countries [16]. A study on Bavarian birth data could not show a statistically significant effect of the two lockdown periods 2020 on preterm births [17]. A special local situation is given in our study due to the fact that, in Munich, the first German COVID-19 infection was detected at the end of January 2020 [18]. This might have led to greater fear of disease and to a good compliance respecting measures during the first lockdown in this region. Consequently, we analyzed the preterm birth rate during the first COVID-19 LockdownPeriod in spring 2020 in the Munich University Perinatal Center.

## Methods

All birth data from the years 2018 to 2020 at the Perinatal Center of the LMU University Hospital, Munich, Germany, were extracted from the obstetric medical record system (View Point Fetal Database 5, GE Health Care, USA). Data were cleaned, infants born prior to 22 + 0 weeks of gestation were excluded from the analysis. The Center, where annually about 4000 births take place, comprises 2 hospitals, both located in the city of Munich. Each location is a tertiary center including a same-level neonatology department.

**Fig. 1:**
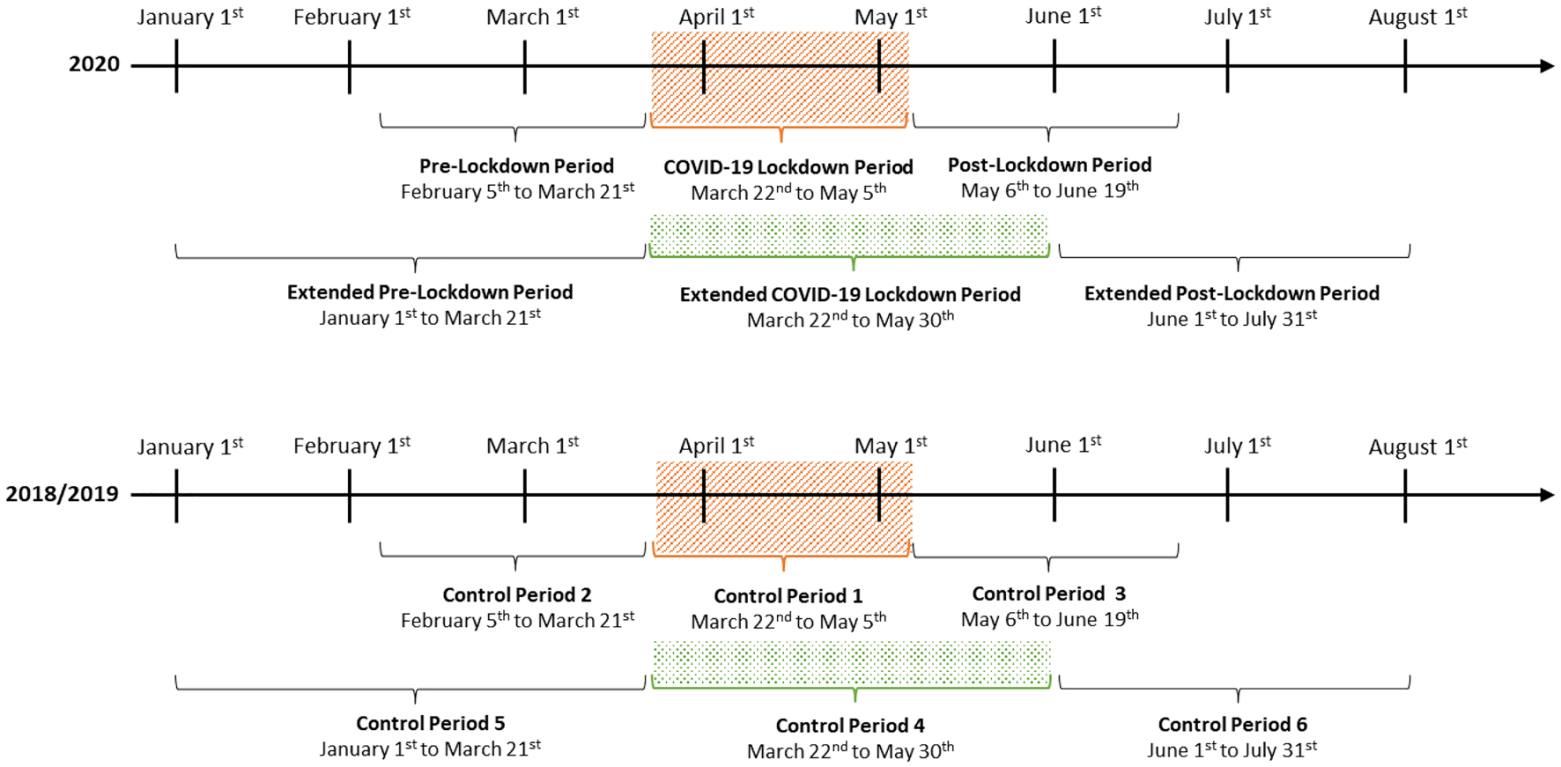
Time periods under study

We analyzed births during the first Bavarian COVID-19 Lockdown Period in 2020 and compared these data to the combined birth outcomes from the same period of the preceding 2 years 2018 and 2019. Because life went back to normal very slowly after the first lockdown, we additionally examined an Extended COVID-19 Lockdown Period in 2020, including four additional weeks and compared those with the combination of the equivalent time periods in 2018 and 2019 (Fig. 1). The following time periods were analyzed and labeled as indicated:

COVID-19 Lockdown Period: March 22nd to May 5th, 2020.

Control Period 1: March 22nd to May 5th, 2018, and 2019.

Extended COVID-19 Lockdown Period: March 22nd to May 30th, 2020.

Control Period 4: March 22nd to May 30th, 2018, and 2019.

To precisely evaluate lockdown effects, we controlled for changes in defined time intervals before and after the lockdown periods, always comparing the time intervals of the year 2020 to the corresponding combined periods in the preceding 2 years. We varied these time intervals in length, first analyzing a time interval before and after the actual lockdown equivalent in days to the duration of the first lockdown, second analyzing longer intervals before and after an extended lockdown period (Fig. 1).

Pre-Lockdown Period: February 5th to March 21st, 2020.

Control Period 2 Pre-Lockdown: February 5th to March 21st, 2018, and 2019.

Post-Lockdown Period: May 6th to June 19th, 2020.

Control Period 3 Post-Lockdown: May 6th to June 19th, 2018, and 2019.

Extended Pre-Lockdown Period: January 1st to March 21st, 2020.

Control Period 5 Extended Pre-Lockdown: January 1st to March 21st, 2018, and 2019.

Extended Post-Lockdown Period: June 1st to July 31st, 2020.

Control Period 6 Extended Post-Lockdown: June 1st to July 31st, 2018, and 2019.

To evaluate the preterm birth rate in a single perinatal unit, possible short-term hospital capacity changes must be considered. According to state data, there were no changes in the number of NICU beds in all 5 tertiary perinatal centers in Munich, the number of beds was constantly 74 in the years under study [19]. With the help of a centralized capacity monitoring system that has been in use in Munich since 2013, it was possible to control for reduced admission rates due to a shortage in beds or staff. This interdisciplinary medical care capacity management system (IVENA eHealth, mainis IT, Frankfurt, Germany) displays the current health-care capacities in the whole city of Munich to the dispatchers of the emergency medical services (EMS) command center who assign rescue vehicles to a receptive hospital. Through IVENA, hospitals can indicate lacking capacity in a special field. In obstetrics, four categories can be signed out individually: 23–29 weeks of pregnancy; 30–32 weeks of pregnancy; 33–36 weeks of pregnancy, and ≥ 37 weeks of pregnancy. If the Neonatal Intensive Care Unit is occupied, the hospital can be closed for emergencies in early pregnancy weeks. We analyzed time in minutes per month when our center was unable to accept further pregnant patients during the COVID-19 lockdown period 2020 compared to the same time in 2019. Numbers of nursing and medical staff as well as the leading personal remained unchanged throughout the whole 3-year period.

For descriptive and statistical analysis, we used SPSS Statistics 28 program (IBM, Chicago, IL, USA). Continuous variables are presented as mean with standard deviation. Categorical variables are shown as numbers and percentages. To compare groups, Student’s *t* test, Mann–Whitney *U* test, Fisher’s exact test, and Chi-squared tests were used. A *p* value below 0.05 was considered statistically significant. Logistic regression was used to calculate odds ratios with 95% confidence intervals.

Primary outcomes were the rate of preterm born infants and preterm births related to the total number of births. Secondary outcomes were the rate of preterm born children in different pre- and post-COVID-19 groups in relation to the corresponding control groups, as well as the rate of stillbirths, mode of delivery, intended mode of delivery, rates of multiple pregnancies, rates of preterm multiple pregnancies, adverse newborn outcomes (APGAR 5 min < 5 and/or arterial umbilical cord blood-pH < 7), and birth weight in the respective comparison groups. Stillbirth was defined when intrauterine fetal death (IUFD) had occurred after 22 weeks of pregnancy.

## Results

A total of 4077 children were born in 2018, 4157 in 2019, and 3664 in 2020. Mother’s mean age was 32.9 years with a minimum of 14 and maximum of 55 years. There was no statistically significant difference in mother’s age between the years studied. The Cesarean section rate was similar in all 3 years (30.4% in 2018, 29.6% in 2019, and 28.6% in 2020), as was the percentage of preterm infants (21.4% in 2018, 20.1% in 2019, and 19.7% in 2020). The rate of multiple pregnancies did not differ significantly between the years (8.1% in 2018, 6.9% in 2019, and 7% in 2020), neither did the mode of delivery (Table 1).

**Table 1:** Baseline characteristics

	2018	2019	2020	
	*N* (%)	*N* (%)	*N* (%)	*P*
Infants per year (including IUFD und late TOP) Singletons Multiples Twins Triplets	4077 3746 (91.9) 331 (8.1) 310 21	4157 3871 (93.1) 286 (6.9) 286 0	3664 3407 (93.0) 257 (7.0) 284 9	0.064
Mother ‘s age (y) mean ± STD (all) Min. (y) / Max. (y)	32.92 ± 5.22 16/52	32.87 ± 5.05 14/55	32.93 ± 4.98 15/51	
Mode of delivery (livebirth, > 22 + 0) Vaginal birth Cesarean section Primary planned C-section	2822 (69.6) 1234 (30.4) 519 (12.7)	2910 (70.4) 1223 (29.6) 485 (11.7)	2620 (71.4) 1040 (28.6) 452 (12.3)	0.169 0.330
Preterm infants (22 + 0 – 36 + 6, live births)	867 (21.4)	831 (20.1)	717 (19.7)	0.175

The Cesarean section rate was similar in the Lockdown Period and the Control Period 1, and there was no difference in the rate of vaginally intended births between the 3 study years. The adverse outcome of the newborns with reference to APGAR < 5 at 5 min and/or arterial umbilical cord blood-pH < 7 was equal during the Lockdown Period and the Control Period 1. The mean birthweight of all infants was statistically significantly higher during the Lockdown Period with a mean of 3263 g in comparison to 3,186 g in Control Period 1 (*p* = 0.014). There were less preterm births in the COVID-19 Lockdown Period (17.9%) compared to the same time intervals in the 2 years before (20.4%), though this difference does not reach statistical significance (*p* = 0.158) (Table 2).

**Table 2:** Maternal and infants characteristics in COVID-19 Lockdown Period and Control Period 1

	COVID-19 Lockdown Period March 22nd to May 5th, 2020	Control Period 1 March 22nd to May 5th, 2018, and 2019	
	*N* (%)	*N* (%)	*P*
Mother’s age (y), mean ± STD	32.62 ± 5.12	32.92 ± 5.18	0.373
Births	266	1,090	
Infants (livebirth > 22 + 0 wks) Singletons Multiples	462 447 (96.8) 15 (3.2)	1,000 923 (92.3) 77 (7.7)	** < 0.001**
Gestational age (weeks.days), mean ± STD	38.56 ± 2.43	38.39 ± 2.47	0.234
Birthweight (g) mean ± STD	3,263.3 ± 613.9	3,186.5 ± 629.5	**0.014**
< 1500 g	10 (2.2)	16 (1.6)	0.287
< 2500 g	41 (8.9)	118 (11.8)	0.055
Preterm births (< 37 + 0)	78 (17.9)	188 (20.4)	0.158
Preterm infants (< 37 + 0) Singletons Multiples	86 (18.6) 75 (87.2) 11 (12.8)	232 (23.2) 165 (71.1) 67 (28.9)	**0.027** **0.003**
Mode of delivery (children) Vaginal birth C-section Primary planned C-section	333 (72.1) 129 (27.9) 57 (12.3)	704 (70.4) 296 (29.6) 110 (11.0)	0.536 0.479
Mode of delivery in preterms Vaginal birth C-section Primary planned C-section	39 (45.3) 47 (54.7) 16 (18.6)	113 (48.7) 119 (51.3) 32 (13.8)	0.615 0.294
Mode of delivery in preterm multiples Primary planned C-section	6 (54.4)	16 (23.9)	0.06
Adverse newborn outcome (APGAR 5 min < 5 and/or arterial pH < 7)	3 (0.7)	7 (0.7)	1.00

Bold values indicate statistically significant values (*p*< 0.05)

**Fig. 2:**
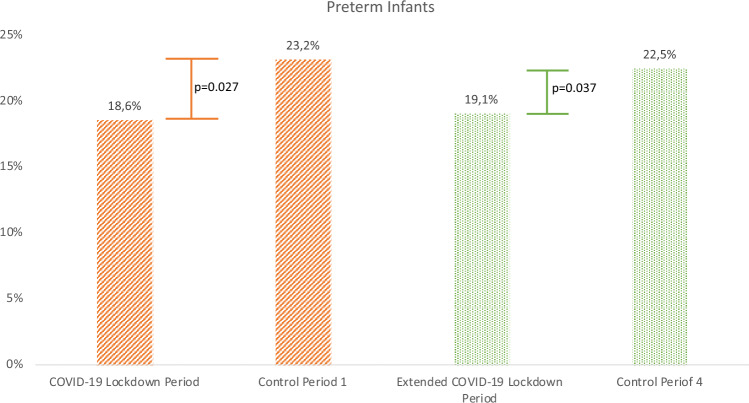
Statistically significant number of preterm infants in the COVID -19 Lockdown Period / Extended COVID-19 Lockdown Period compared with respective control periods; bars reflect the percentage of preterm infants of the distinctive time period.

Analyzing data based on the infants, we could show that in the COVID-19 Lockdown Period, 18.6% of the infants were born preterm as compared to 23.2% during the Control Period 1. This result corresponds to a significant risk reduction for being born preterm of 25% during the first Lockdown Period (OR = 0.757; [95% KI: 0.574; 0.998]; *p* = 0.027) (Table 2, Fig. 2).

This effect remains statistically significant prolonging the analysis to the Extended COVID-19 Lockdown Period (March 22 to May 30, 2020). During this time, 19.1% of all infants were born preterm as compared to 22.5% in the corresponding Control Period 4 (March 22 to May 30, 2018, and 2019) (*p* = 0.037) Fig. 2). In the 6 weeks after the COVID-19 Lockdown Period (Post-Lockdown Period, May 6 to June 19th, 2020) compared to Control Period 3 (May 6 to June 19th, 2018, and 2019) a slightly higher rate of preterm children was delivered without statistical significance (23.6% vs. 20.8%, *p* = 0.111). However, in the Extended Post-Lockdown Period (June 1st to July 31, 2020), the rate of preterm born children equalized with 22.1% in both groups (Table 3).

**Table 3:** Preterm infants in varying periods

	*N* (%)	*N* (%)	*P*
	**COVID-19 Lockdown Period** March 22nd to May 5th, 2020	**Control Period 1** March 22nd to May 5th, 2018, and 2019	
Preterm infants < 37 + 0	86 (18.6)	232 (23.2)	**0.027**
	**Pre-Lockdown Period** February 5th to March 21st, 2020	**Control Period 2 Pre-Lockdown** February 5th to March 21st, 2018, and 2019	
Preterm infants < 37 + 0	88 (17.6)	186 (18.6)	0.355
	**Post-Lockdown Period** May 6th to June 19th, 2020	**Control Period 3 Post-Lockdown** May 6th to June 19th, 2018, and 2019	
Preterm infants < 37 + 0	124 (23.6)	224 (20.8)	0.111
	**Extended COVID-19 Lockdown Period** March 22nd to May 30th, 2020	**Control Period 4** March 22nd to May 30th, 2018, and 2019	
Preterm infants < 37 + 0	144 (19.1)	359 (22.5)	**0.037**
	**Extended Pre-Lockdown Period** January 1st to March 21st, 2020	**Control Period 5 Extended Pre-Lockdown** January 1st to March 21st, 2018, and 2019	
Preterm infants < 37 + 0	158 (18.2)	343 (19.3)	0.271
	**Extended Post-Lockdown Period** June 1st to July 31st, 2020	**Control Period 6 Extended Post-Lockdown** June 1st to July 31st, 2018, and 2019	
Preterm infants < 37 + 0	153 (22.1)	319 (22.1)	0.521

Bold values indicate statistically significant values (*p*< 0.05)

**Fig. 3:**
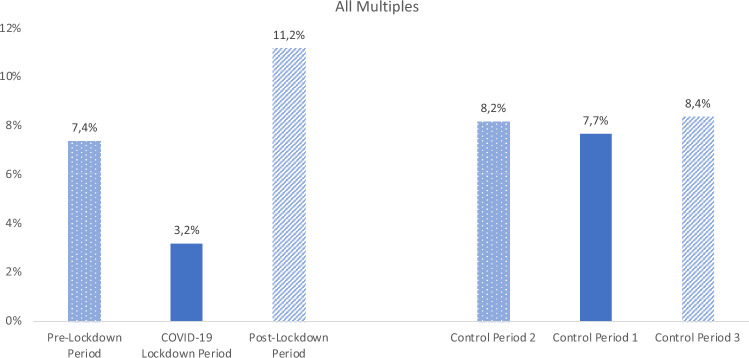
Comparison of the percentage of multiples in different periods on the left: Pre-Lockdown 2020, COVID-19 Lockdown 2020, and Post-Lockdown 2020 which shows a significant dip in the lockdown period with a compensation post-lockdown. On the right, the respective control periods in 2018 and 2019 which show no interperiod difference. Bars reflect the percentage of multiples in the distinctive time periods.

Interestingly, in the COVID-19 Lockdown Period, the rate of births from multiple pregnancies (preterm and mature neonates) was only 3.2% compared to 7.7% in the Control Period 1 (*p* < 0.001). This effect was reversed after the first lockdown, in the Post-Lockdown Period in 2020. In the weeks after the Lockdown in 2020, the percentage of infants born from multiple pregnancies more than tripled (Fig. 3). Compared to the Control Period 3, more children from multiple pregnancies (all weeks) were born in 2020 (11.2% in 2020 vs. 8.4% in the Control Period 3, *p* = 0.067). In the Pre-Lockdown Period in 2020 (February 5th to March 21st, 2020), no difference in the number of multiples could be found compared to the respective control interval (Table 4).

**Table 4:** Characteristics of multiples in COVID-19 Lockdown Period and Control Period 1

	*N* (%)	*N* (%)	*P*
	**COVID-19 Lockdown Period** March 22nd to May 5th, 2020	**Control Period 1** March 22nd to May 5th, 2018, and 2019	
Multiples all livebirths	15 (3.2)	77 (7.7)	** < 0.001**
	**Pre-Lockdown Period** February 5th to March 21st, 2020	**Control Period 2 Pre-Lockdown** February 5th to March 21st, 2018, and 2019	
Multiples all livebirths	37 (7.4)	82 (8.2)	0.614
	**Post-Lockdown Period** May 6th to June 19th, 2020	**Control Period 3 Post-Lockdown** May 6th to June 19th, 2018, and 2019	
Multiples all livebirths	59 (11.2)	90 (8.4)	0.067

Bold values indicate statistically significant values (*p*< 0.05)

Accordingly, during the COVID-19 Lockdown Period, 12.8% of the preterm newborns were multiples compared to 28.9% in Control Period 1 (*p* = 0.003) (Table 2). Considering preterm infants from singleton pregnancies alone, we could not show a statistical significant difference between the different periods (17.1% preterm infants in the Lockdown Period, 18.0% in the Control Period 1, *p* = 0.385).

Modes of delivery across preterm singletons or multiples did not differ regarding vaginal births and Cesarean sections between the COVID-19 Lockdown Period compared to the Control Period 1 (*p* = 0.615). Nonetheless, the reduction of infants born preterm during the Lockdown Period 2020 was accompanied by a reduction of spontaneous onset of labor in preterm births. The rate of elective Cesarean sections in preterm infants was higher during the Covid-19 Lockdown Period in comparison to the Control Period 1 (18.6% vs. 13.8%; *p* = 0.294). For preterm multiples, this difference was more pronounced almost reaching statistical significance (23.9% elective Cesarean sections during Control Period 1 and 54.5% elective Cesarean sections during the COVID-19 Lockdown Period *p* = 0.065) (Table 2).

During the COVID-19 Lockdown Period, no difference in the IUFD rate in relation to the Control Period 1 was detected (0.9% vs. 0.7% in Control Period 1). Interestingly, after the liberalization of lockdown restrictions and with lower incidence of COVID-19 infections, the IUFD rate tended to rise in the Post-Lockdown Period (June 1st to July 31st, 2020) as compared to the Control Period 6 (0.9% vs. 0.5%; *p* = 0.068) (Table 5).

**Table 5:** IUFD in varying periods; all probabilities

	*N* (%)	*N* (%)	*P*
	**COVID-19 Lockdown Period** March 22nd to May 5th, 2020	**Control Period 1** March 22nd to May 5th, 2018, and 2019	
IUFD	4 (0.9)	7 (0.7)	0.750
	**Pre-Lockdown Period** February 5th to March 21st, 2020	**Control Period 2 Pre-Lockdown** February 5th to March 21st, 2018, and 2019	
IUFD	3 (0.6)	6 (0.6)	1.00
	**Post-Lockdown Period** May 6th to June 19th, 2020	**Control Period 3 Post-Lockdown** May 6th to June 19th, 2018, and 2019	
IUFD	1 (0.2)	5 (0.5)	0.670
	**Extended COVID-19 Lockdown Period** March 22nd to May 30th, 2020	**Control Period 4** March 22nd to May 30th, 2018, and 2019	
IUFD	4 (0.5)	12 (0.7)	0.789
	**Extended Pre-Lockdown Period** January 1st to March 21st, 2020	**Control Period 5 Extended Pre-Lockdown** January 1st to March 21st, 2018, and 2019	
IUFD	3 (0.3)	13 (0.7)	0.178
	**Extended Post-Lockdown Period** June 1st to July 31st, 2020	**Control Period 6 Extended Post-Lockdown** June 1st to July 31st, 2018, and 2019	
IUFD	6 (0.9)	4 (0.3)	0.088

The capacity monitoring system (IVENA) did not show any capacity reduction during the COVID-19 Lockdown Period, neither for obstetrics nor for neonatology. With regard to capacity shut down, no difference could be found in any of the analyzed sign-out sub-groups (Table 6).

**Table 6:** Additive sign-out time in the IVENA system of both hospitals in April 2019 and April 2020

Sign-out time in minutes (IVENA system)	April 2019	April 2020	*P* value
Complete sign-out time	276,520	289,280	1.00
Obstetrics 23—29 week of pregnancy	76,090	85,520	1.00
Obstetrics 30 – 32 week of pregnancy	76,210	85,520	1.00
Obstetrics 33 – 36 week of pregnancy	81,810	83,360	1.00
Obstetrics > 37 week of pregnancy	42,410	34,880	0.937

## Discussion

In this study, we confirm a decreased number of preterm infants born during the COVID-19 Lockdown Period, comparable to data shown in other studies, especially from high-income countries [4–6, 8]. The reasons for this decrease of preterm deliveries during the COVID-19 Lockdown Period are still unclear, but several explanations are postulated. Among others, reduced working hours, reduced physical and/or emotional stress related to work, fewer infections (better hygiene and fewer social interactions), less smoking and drug use, being at home with support of the family, having time for exercise, and reduced exposure to environmental pollutants, less car driving (less stress and fewer accidents) could all be possible reasons [8]. Overall, this could have led to a feeling of less daily stress. Especially in the study area, lockdown measures and contact restrictions were strictly enforced and taken seriously because the first German COVID-19 case was detected in the Munich area [16].

The decrease in preterm birth rates in our cohort was predominantly caused by a decrease of preterm births in multiple pregnancies. This focus on preterm births in multiple pregnancy during COVID-19 Lockdown is one strength of our study. Most of the other international studies excluded multiple pregnancies in their calculations [4, 5, 20].Thus, this novel observation of our study may help to understand the mechanisms driving impact of lockdown measures on the prevalence of premature birth. Preterm births in twins, more than in singletons, may predominantly be caused mechanically by early contractions, followed by cervical insufficiency finally leading to preterm birth. In addition, we identified more twin births in the Post-Lockdown period in 2020 in comparison to the same period in the preceding years. This indicates that the neonates that were not born preterm during the lockdown period were born later and thus less likely to be preterm. As in other studies, the reduction in preterm births was accompanied by a reduction in spontaneous preterm births [16, 21]. Therefore, our results point at a reduction in physical activity and daily stressors as reason for the protective effect of lockdown measures.

According to our data, published data from the Bavarian medical quality control system (BQS) showed unadjusted significantly lower preterm birth rates in Lockdown Period 2020 for singleton pregnancies (5.71% vs. 6.41%; OR 0.88; *p* < 0.001). However, these effects could not be confirmed after adjusting for long-term trends (adj. OR = 0.99; *p* = 0.73). For twin pregnancies, lower preterm birth rates during lockdown could be demonstrated without statistical significance (52.99% vs. 56.26%; adj.p = 0.31). These region-wide dataset [17] further supports that our data show a real local effect and not just a shift in preterm births to other hospitals in the area.

Besides the preterm born infants, an evaluation of stillbirth rates in the study group during the COVID-19 Lockdown Period compared to the respective time intervals in 2018 and 2019 was made. No difference could be seen in stillbirth rates between the COVID-19 Lockdown Period compared to Control Period 1 in 2018 and 2019. These results corroborate findings from other studies conducted in high-income countries, which also did not detect an increase in the stillbirth rate during the first COVID-19 Lockdown Period [12–14, 19, 20]. In contrast to that, the analysis of the Bavarian perinatal survey of the first COVID-19 Lockdown Period showed higher stillbirth rates than in the corresponding periods from 2010 to 2019. However, the effects on the stillbirth rate in this study could no longer be observed after adjustment for seasonal and long-term trends [22]. Interestingly, after the liberalization of COVID-19 restrictions and when lower incidences of COVID-19 infections occurred later after the COVID-19 Lockdown Period (June 1st to July 31st, 2020), the rate of stillbirths tended to rise in the present study compared to the control periods 2018 and 2019. One possible explanation could be that pregnant women avoided routine appointments during COVID-19 Lockdown, and therefore fetal risks for perinatal death were not detected early enough. Another theory for this finding could be asymptomatic SARS-CoV-2 infections in these stillbirth events. It is described that especially SARS-CoV-2 infection in early pregnancy could lead to placental pathology like fetal vascular malperfusion [23]. Thus, potentially asymptomatic SARS-CoV-2 infection in earlier pregnancy might be the reason for the higher stillbirth rate in the Extended Post-Lockdown Period 10–18 weeks after the first COVID-19 Lockdown. In the German CRONOS register study, an increased risk of stillbirth in patients after SARS-CoV-2 infection in early pregnancy could be shown [24].

The single-center setup of the study can be seen as a potential limitation as it might not be representative for all obstetrical units in Germany. However, an advantage of our analysis in this single center with over 4000 births annually lies in the possibility to look deeper into the details of the patients and to be aware of the surrounding confounders and resources during the time intervals studied. A further strength of our study is that we include multiple pregnancies. We also provide an external control by the city-wide capacity system, which showed no difference with regard to closure of the unit during the lockdown, so an artificial reduction of preterm births due to logistical reasons can be ruled out. Besides, Bavaria-wide data do not show a rise of preterm births in any other nearby hospital.

## Conclusion

The present analysis demonstrates a statistically significant reduction in the rate of preterm children and preterm deliveries in a German perinatal center during the COVID-19 Lockdown Period in 2020 compared to control periods in 2018 and 2019. The results show a dominant reduction of preterm births in multiple pregnancies. We postulate that stress reduction and less activity might have led to a protective effect by lockdown measures. There was no increase in the stillbirth rate during the Lockdown Period.

## Data Availability

Not applicable.

## Abbreviations

BQS: Bavarian obstetric quality parameter
COVID-19: Coronavirus disease 2019
SARS-CoV-2: Severe acute respiratory syndrome coronavirus 2
EMS: Emergency medical services
IUFD: Intrauterine fetal death
IVENA: Interdisciplinary medical care capacity management system (“Interdisziplinärer Versorgungsnachweis”)
NICU: Neonatal intensive care unit

## References

[1] Sohrabi C, Alsafi Z, O'Neill N, Khan M, Kerwan A, Al-Jabir A (2020). World health organization declares global emergency: a review of the 2019 novel coronavirus (COVID-19). Int J Surg.

[2] Pecks U, Kuschel B, Mense L, Oppelt P, Rüdiger M (2020). Pregnancy and SARS-CoV-2 infection in Germany-the CRONOS registry. Dtsch Arztebl Int.

[3] Roberton T, Carter ED, Chou VB, Stegmuller AR, Jackson BD, Tam Y (2020). Early estimates of the indirect effects of the COVID-19 pandemic on maternal and child mortality in low-income and middle-income countries: a modelling study. Lancet Glob Health.

[4] Been JV, Burgos Ochoa L, Bertens LCM, Schoenmakers S, Steegers EAP, Reiss IKM (2020). Impact of COVID-19 mitigation measures on the incidence of preterm birth: a national quasi-experimental study. Lancet Public Health.

[5] Hedermann G, Hedley PL, Bækvad-Hansen M, Hjalgrim H, Rostgaard K, Poorisrisak P (2021). Danish premature birth rates during the COVID-19 lockdown. Arch Dis Child Fetal Neonatal Ed.

[6] Rolnik DL, Matheson A, Liu Y, Chu S, McGannon C, Mulcahy B (2021). Impact of COVID-19 pandemic restrictions on pregnancy duration and outcome in Melbourne, Australia. Ultrasound Obstet Gynecol.

[7] Kc A, Gurung R, Kinney MV, Sunny AK, Moinuddin M, Basnet O (2020). Effect of the COVID-19 pandemic response on intrapartum care, stillbirth, and neonatal mortality outcomes in Nepal: a prospective observational study. Lancet Glob Health.

[8] Berghella V, Boelig R, Roman A, Burd J, Anderson K (2020). Decreased incidence of preterm birth during coronavirus disease 2019 pandemic. Am J Obstet Gynecol MFM.

[9] Leibovitch L, Reichman B, Mimouni F, Zaslavsky-Paltiel I, Lerner-Geva L, Wasserteil N (2021). Preterm singleton birth rate during the COVID-19 lockdown: a population-based study. Am J Perinatol.

[10] Alshaikh B, Cheung PY, Soliman N, Brundler MA, Yusuf K (2022). Impact of lockdown measures during COVID-19 pandemic on pregnancy and preterm birth. Am J Perinatol.

[11] Yalçin SS, Boran P, Tezel B, Şahlar TE, Özdemir P, Keskinkiliç B (2022). Effects of the COVID-19 pandemic on perinatal outcomes: a retrospective cohort study from Turkey. BMC Pregnancy Childbirth.

[12] Khalil A, von Dadelszen P, Draycott T, Ugwumadu A, O'Brien P, Magee L (2020). Change in the incidence of stillbirth and preterm delivery during the COVID-19 pandemic. JAMA.

[13] Pasternak B, Neovius M, Söderling J, Ahlberg M, Norman M, Ludvigsson JF (2021). Preterm birth and stillbirth during the COVID-19 pandemic in Sweden: a nationwide cohort study. Ann Intern Med.

[14] Arnaez J, Ochoa-Sangrador C, Caserío S, Gutiérrez EP, Jiménez MDP, Castañón L (2021). Lack of changes in preterm delivery and stillbirths during COVID-19 lockdown in a European region. Eur J Pediatr.

[15] Handley SC, Mullin AM, Elovitz MA, Gerson KD, Montoya-Williams D, Lorch SA (2021). Changes in preterm birth phenotypes and stillbirth at 2 Philadelphia hospitals during the SARS-CoV-2 pandemic, March-June 2020. JAMA.

[16] Chmielewska B, Barratt I, Townsend R, Kalafat E, van der Meulen J, Gurol-Urganci I (2021). Effects of the COVID-19 pandemic on maternal and perinatal outcomes: a systematic review and meta-analysis. Lancet Glob Health.

[17] Stumpfe FM, Schneider MO, Hein A, Faschingbauer F, Kehl S, Hermanek P (2022). Limited effects of SARS-CoV-2 pandemic-related lockdowns and reduced population mobility on preterm birth rates: a secondary analysis of Bavarian obstetric quality parameters from 2010 to 2020. Geburtshilfe Frauenheilkd.

[18] Rothe C, Schunk M, Sothmann P, Bretzel G, Froeschl G, Wallrauch C (2020). Transmission of 2019-nCoV infection from an asymptomatic contact in Germany. N Engl J Med.

[19] Pflege BSfGu. Krankenhausplan des Freistaates Bayern. Ausgabe 44 (112019), Ausgabe 45 (112020), Ausgabe 46 (112021); https://digital.zlb.de

[20] Philip RK, Purtill H, Reidy E, Daly M, Imcha M, McGrath D (2020). Unprecedented reduction in births of very low birthweight (VLBW) and extremely low birthweight (ELBW) infants during the COVID-19 lockdown in Ireland: a ‘natural experiment’ allowing analysis of data from the prior two decades. BMJ Glob Health.

[21] Melov SJ, Elhindi J, McGee TM, Lee VW, Cheung NW, Chua SC (2022). Investigating service delivery and perinatal outcomes during the low prevalence first year of COVID-19 in a multiethnic Australian population: a cohort study. BMJ Open.

[22] Stumpfe FM, Schneider MO, Antoniadis S, Mayr A, Fleckenstein T, Staerk C (2022). Lack of evidence for effects of lockdowns on stillbirth rates during the SARS-CoV-2 pandemic in Bavaria: analysis of the Bavarian perinatal survey from 2010 to 2020. Arch Gynecol Obstet.

[23] Resta L, Vimercati A, Cazzato G, Mazzia G, Cicinelli E, Colagrande A (2021). SARS-CoV-2 and placenta: new insights and perspectives. Viruses.

[24] Iannaccone A, Mand N, Schmidt B, Rüdiger M, Reisch B, Pecks U (2022). Is the risk of still and preterm birth affected by the timing of symptomatic SARS-CoV-2 infection during pregnancy? Data from the COVID-19 Related Obstetrics and Neonatal Outcome Study Network. Germany. Am J Obstet Gynecol..

